# Resveratrol, Rapamycin and Metformin as Modulators of Antiviral Pathways

**DOI:** 10.3390/v12121458

**Published:** 2020-12-17

**Authors:** Francesca Benedetti, Vincenzo Sorrenti, Alessandro Buriani, Stefano Fortinguerra, Giovanni Scapagnini, Davide Zella

**Affiliations:** 1 Institute of Human Virology, Department of Biochemistry and Molecular Biology, University of Maryland School of Medicine, Baltimore, MD 21201, USA; fbenedetti@ihv.umaryland.edu; 2 Department of Pharmaceutical and Pharmacological Sciences, University of Padova, 35131 Padova, Italy; vincenzo.sorrenti@unipd.it; 3 Bendessere™ Study Center, Via Prima Strada 23/3, 35129 Padova, Italy; 4 Maria Paola Belloni Center for Personalized Medicine, Data Medica Group (Synlab Limited), 35100 Padova, Italy; alessandro.buriani@gmail.com; 5 IRCCS SDN, 80143 Napoli, Italy; stefano.fortinguerra@gmail.com; 6 Department of Medicine and Health Sciences “V. Tiberio”, University of Molise, 86100 Campobasso, Italy

**Keywords:** resveratrol, rapamycin, metformin, antiviral, dietary supplement

## Abstract

Balanced nutrition and appropriate dietary interventions are fundamental in the prevention and management of viral infections. Additionally, accurate modulation of the inflammatory response is necessary to achieve an adequate antiviral immune response. Many studies, both in vitro with mammalian cells and in vivo with small animal models, have highlighted the antiviral properties of resveratrol, rapamycin and metformin. The current review outlines the mechanisms of action of these three important compounds on the cellular pathways involved with viral replication and the mechanisms of virus-related diseases, as well as the current status of their clinical use.

## 1. Resveratrol

Resveratrol (trans-3,4,5-trihydroxystilbene) is a small polyphenol natural molecule that can be obtained from several sources, including red wine, grapes, a variety of berries, peanuts and certain medicinal plants [1,2]. With lipophilic characteristics that make it easily absorbed, metabolized and excreted, resveratrol is used as a dietary supplement, even though it has a low bioavailability that limits its use [3,4,5]. Indeed, the way it is consumed, and the kind of food ingested influence its absorption [6]. Upon oral administration, resveratrol complexes with membrane transporters, which allow its absorption by passive diffusion, eventually ending up in the bloodstream, where it can be detected in three forms: glucuronide, sulfate and free [6]. The latter form is typically associated with albumin or lipoproteins, to allow for better circulation and entry in the cells [7]. Further metabolism of resveratrol takes place in the liver, producing different metabolites that can be detected in the urine [8].

Both in vitro experiments with mammalian cells and in vivo experiments with small animal models have shown a number of positive effects correlated with the use of resveratrol [9,10,11,12], including reduction of inflammation [13], antioxidant and antiaging [14,15,16], and anticancer [17,18,19,20,21]. For this reason, resveratrol is considered a promising molecule for the prevention and treatment of a number of conditions, including autoimmune disorders, cardiovascular and neurodegenerative diseases as well as some chronic diseases [22,23,24,25,26]. In addition, resveratrol was demonstrated to extend the lifespan of several evolutionarily distant species, including *Saccharomyces cerevisiae* [27], *Caenorhabditis* *elegans* [28] and *Drosophila melanogaster* [29], thus further confirming the conserved nature of the pathways involved.

A number of in vivo studies have provided the rationale for the use of the polyphenolic molecule resveratrol in several humans disorders. In particular, inhibition of important gene pathways like the NF-κB pathway supports its use as an antioxidant, while inhibitions of viral replication, protein synthesis, gene expression and nucleic acid synthesis support its use as an antiviral [30]. In fact, resveratrol is considered a molecule with promising immunomodulatory effects which is able to regulate immunological and inflammatory responses. 

A number of human viruses seem to be sensitive to the effects of resveratrol (Table 1), including influenza virus [31], respiratory syncytial virus [32,33,34,35,36,37], varicella zoster virus [38], Epstein-Barr virus [39,40], herpes simplex virus [41,42,43,44], human immunodeficiency virus [45,46,47] and enterovirus [48,49]. While in these cases, treatment with resveratrol reduced viral infection, a notable exception was an in vitro model of Hepatitis C where infection was enhanced [50].

Using the mammalian MDCK cells normally used for influenza research as in vitro model, Palamara et al. (2005) showed that resveratrol treatment resulted in inhibition of viral nucleoproteins translocation to the nucleus, and that this diminished the inhibitory effect of protein kinase C related pathways. The effect was not correlated with the glutathione-mediated antioxidant activity of resveratrol [31].

Regarding respiratory syncytial virus (RPSV) infection, a disease of the respiratory system, in vivo treatment of mice (injected intraperitoneally with resveratrol 30 mg/Kg/day for 5 days) resulted in amelioration of inflammatory symptoms, likely because of modulation of several molecules, including induction of muscarinic 2 receptor (M2R), inhibition of toll/IL-1R domain-containing adaptor inducing IFN (TRIF) signaling and reduction of toll-like receptor 3 expression [36]. In yet another set of experiments in mice (injected intraperitoneally with resveratrol 30 mg/Kg/day for five days), experimental data showed decreased IFN-*γ* expression accompanied by reduced airway inflammation and decreased hyper-responsiveness (AHR). A number of other proteins were also affected, including: (i) increased expression of sterile-*α*- and armadillo motif-containing protein (SARM), (ii) decreased expression of matrix metalloproteinase 12 (MMP-12) and, (iii) decreased TIR-domain-containing adapter-inducing interferon-*β* (TRIF) [33,51]. Additionally, in vitro studies, where resveratrol was used to treat epithelial cells infected with RPSV, showed reduction in both production of interleukin- (IL-) 6 and in viral replication. Moreover, hampering of the expression of viral induced toll-like receptor domain and TANK binding kinase 1 (TBK1) protein [34] was observed. Finally, resveratrol in combination with a baicalin (a flavonoid found in numerous species of *Scutellaria*) joint enema (resveratrol administered 30 mg/Kg/day retention enema for 30 min) increased the antiviral mechanisms in mice infected with RPSV, namely increasing the levels of IL-2, IFN-*γ*, and tumor necrosis factor-alpha (TNF-*α*) [32]. 

With respect to Varicella Zooster Virus (VZV), resveratrol noticeably decreased the synthesis of intermediate early protein (IE 62) in MRC-5 cells (human lung cells line) early in infection (within 30 h) [38].

Resveratrol inhibited early antigen production of Epstein-Barr virus (EBV) in infected B-cells (Raji) [53]. Together with decreasing EBV lytic cycle by reducing transcription genes and proteins, (Rta, Zta, and diffused early antigen, EA-D), as well as hampering the activity by EBV immediate-early proteins (BRLF1 and BZLF1 promoters), resveratrol reduced the production of viral particles [40]. These data confirmed early findings showing dose-dependent inhibition by resveratrol of expression of lytic genes and viral particles, where its major antiviral mechanism was associated with (i) inhibition of transcription factors NF-κB and AP1; (ii) inhibition of protein synthesis, and (iii) reduction in ROS production [53]. Due to the broad recognition of EBV as an oncogenic virus, resveratrol was employed to prevent transformation of human B-cells caused by EBV. The effect of resveratrol was mainly observed on the downregulation of two antiapoptotic proteins, namely Mc1 and survivin, together with the suppression of signaling induced by NF-κB, STAT-3, miR-155 and miR-34a in EBV infected cells [39]. Finally, resveratrol (at a concentration of 85 nM over the duration of the experiment, administered by topic treatment at the site of skin area where tumorigenesis was promoted) was shown to reduce papilloma production by 60% in EBV-infected mice 20 weeks post inoculation [52].

Treatment with resveratrol resulted in the decreased production of early viral protein ICP-4 of herpes simplex virus-1 and herpes simplex virus-2 (HSV-1 and HSV-2) in Vero cells (kidney cells derived from monkey) and infected human lung cells (MRC-5). Consequently, the viral replication and load was reduced in a dose-dependent and reversible manner [42]. In addition, treatment with resveratrol of latently infected neuron cells deferred the interphase stage of the cell cycle and hindered reactivation of HSVs [43]. In in vivo studies on nude mice, a topical unguent of 12.5% and 15% of resveratrol applied 2–5 times daily for five days on scratched skin infected with HSV-1 inhibited the development of cutaneous lesions [44]. Moreover, a vaginal cream of 19% resveratrol applied topically to mice after local infection with HSV-2 and HSV-1 prevented the development of vaginal lesions with a 3% mortality rate, as opposed to the 37% mortality rate observed in the placebo group [42]. Induction of a quick and continuous release of ROS, resulting in the inhibition of NF-κB and extracellular signal-regulated kinases/mitogen-activated protein kinases (ERK/MAPK), together with a marked reduction in the expression of several HSV genes (immediate-early, early, and late) and viral DNA synthesis were the most likely molecular mechanisms ascribed to these effects induced by resveratrol [41,54].

Resveratrol and its analogs, alone or in combination with decitabine (a nucleoside used for myelodysplastic syndromes), has been employed in vitro (5 μM) to inhibit DNA synthesis of the HIV molecular clone NL4–3 (which harbors the mutant M184V reverse transcriptase (RT)) during the reverse transcription step of the HIV life cycle [45,46]. To further support the possibility of using resveratrol (or its analogues) as adjuvant in anti-HIV preexposure prophylaxis (PrEP) formulation, it was shown that low doses of resveratrol (and its chemical relative pterostilbene) were able to block HIV-1 infection in resting CD4 T cells, interfering with the step involving reverse transcription [47]. This anti-HIV effect was abrogated in the presence of Vpx, an HIV-1 and simian immunodeficiency virus protein that causes an increase of deoxynucleoside triphosphate (dNTP) levels, or when exogenous deoxynucleosides where added to the culture medium. These findings further confirm the ability of resveratrol to inhibit ribonucleotide reductase and to decrease dNTP levels in cells. Another resveratrol analog, 3,3′,4,4′,5,5′-hexahydroxy-trans-stilbene (M8), was shown to have a potent anti-HIV activity against different viral variants. More in detail, M8 blocked HIV fusion with host cells, indicating an effect at the very early steps of viral entry. Taken together, these latest data indicate that the novel resveratrol derivative, M8, has anti-HIV-1 effects and likely a different mechanism of action than anti-HIV-1 drugs currently in use [55]. 

Resveratrol treatment also inhibited the synthesis of Enterovirus 71 (EV 71) viral protein 1 (VP1) and phosphorylation of proinflammatory cytokines (IKK*α*, IKK*β*, IKK*γ*, IKB*α*, NF-κB p50, and NF-κB p65) in Rhabdomyosarcoma cell line, together with secretion of IL-6 and TNF-*α* [48].

In Vero E6 cells infected by MERS-CoV, resveratrol treatment downregulated the apoptosis induced by viral infection which resulted in increased survival of infected cells, and reduced the expression of nucleocapsid (*N*) protein, essential for MERS-CoV replication [56]. The use of resveratrol has also been proposed in the treatment of patients infected with SARS-CoV-2, due to its ability to decrease the high levels of inflammatory cytokines, such as IL-6 and TNF-α, observed in patients [58,59]. Further data indicating a potential beneficial effect of resveratrol in the treatment of SARS-CoV-2 patients come from the observation that upregulation of Angiotensin Converting Enzyme-2 (ACE-2) has a protective effect on SARS-CoV illness severity, and high intake of resveratrol upregulates ACE-2 receptors [57,60].

A number of molecules have been identified as targets for resveratrol antiviral effects, including regulation of important cellular pathways such as those involved in cell cycle, apoptosis and inflammatory responses [61]. In this regard, resveratrol inhibits the NF-κB pathway through several mechanisms. NF-κB is an important transcription factor, considered a fundamental regulator of the inflammatory cellular response, and it is also involved in cellular proliferation, transformation and tumor development [62,63,64]. Different stimuli, for example, those elicited during bacterial and viral infections, can activate NF-κB, and consequently its role for NF-κB in the innate immune response to infection has been largely described [65]. Resveratrol inhibits the activation of the NF-κB pathway by blocking TNF-α production in human monocyte and macrophage cell lines in vitro and in mouse skin in vivo (topical application of resveratrol 0.25–1 μmol for 4 h) by hampering phosphorylation of IκB by IKK, and as a result, hindering the translocation of NF-κB to the nucleus [66,67,68]. These results shed light on the antiviral effects mediated by resveratrol. In fact, although usually NF-κB activation has a protective role, some viruses rely on NF-κB activation for efficient replication [69], as is the case for influenza A virus [70] and HSV-1 [71]. Consequently, suppression of the NF-κB pathway seems to be one of the mechanisms through which resveratrol exerts its antiviral activity [54]. 

Further emphasizing its role as an anti-inflammatory molecule with potentially useful therapeutically implications, it was observed that resveratrol reduces the expression of several genes with proinflammatory functions, including TNF-α, CCL3, IL-1β in patients with diabetes and with coronary heart diseases [72]. Moreover, resveratrol has the potential to control the levels of inflammation in healthy subjects, since it reduces the expression of a number of other inflammatory and oxidative cytokines and stress markers such as p47phox, CRP, IL-6, JNK1, IKKβ, TLR4 [73,74]. In the emerging field of miRNAs as new signaling molecules, resveratrol seems to exert some anti-inflammatory effects in the monocytoid cell line THP-1, by upregulating miR-663, a miRNA which targets several genes involved in the immune response [75].

A large body of literature emphasizes the effect of resveratrol on enzyme activities or its binding to receptors, activating cellular pathways involved in apoptosis [76,77]. In this regard, it has been shown that upon entry via endocytosis, resveratrol-induced apoptosis follows its accumulation in lipid rafts triggering activation of MAPK and caspases [78]. With a different mechanism, resveratrol has been involved in p53-mediated apoptosis [79,80,81,82], exerted through increased p53-mediated transcriptional activity [83,84,85]. P53 is arguably the most important anticancer protein, considered the “guardian of the genome”, because of its crucial importance in preventing cellular transformation [86]. However, its stimulating effects are counteracted by activation of Sirt1, which has been reported to be one of the main targets of resveratrol [77]; indeed, the activation of Sirt1 may contribute to the beneficial effects of resveratrol in several diseases [87,88,89]. Sirt1 in turn interacts with and deacetylates p53, thus reducing p53-mediated functions [90,91]. Additional studies have highlighted the inhibitory effects of resveratrol on cyclo-oxygenases 1 and 2 (COX 1 and 2), two pro-inflammatory enzymes [92] and its effects on apoptosis [76,93,94]. The apoptosis or survival of viral-infected cells will ultimately depend on the preferential activation by resveratrol of Sirt1 or p53, and on resveratrol’s effects on the NF-κB pathway. 

Another important cellular function influenced by resveratrol is related to cell cycle control. In this regard, by binding the αvβ3 integrin receptor, resveratrol hampers the functions of the IGF-1 receptor, resulting in reduced cells proliferation abilities [95]. A broad list of targets affected by resveratrol includes some cell cycle proteins with nuclear localization, phosphatases and cyclins [96,97]. All these data support the hypothesis that resveratrol is able to limit cellular proliferation. It therefore is not surprising that resveratrol has also been studied for its potential anticancer therapeutic properties. 

Indeed, a number of in vitro data generated in different cancer cell lines, including stomach [98], colorectal [99], intestine [100], liver [101,102,103], kidney [104], blood [105,106,107,108], lung [109,110,111], ovaries [112,113], prostate [114,115], breast [116], glioma cells [117,118,119,120], head and neck tumors [121,122], bone [123], skin [124] and heart [125], as well as experiments in vivo in several animal models [19,126], have shown the anticancer mechanisms of resveratrol [127] and supported its use for cancer prevention due to its direct antitumor effects [128,129,130,131,132]. The route of administration varied from intragastric intubation to intraperitoneal injection, through diet or gavage. The amount also varied, from 10 mg/kg to 100 mg/kg daily, and the time of administration was from a few days to several weeks, indicating the absence of toxicity of resveratrol, but also the need to carefully determine the proper route and level of administration for an effective action [19]. In this regard, resveratrol intervenes in different stages of cellular transformation, affecting pathways usually dysregulated in cancer, including initiation, promotion to progression. In fact, resveratrol has been demonstrated to be involved in cell growth and division, inflammation, apoptosis, metastasis and angiogenesis [21,133]. Though in vivo and in vitro experiments have demonstrated generally positive outcomes, a certain variability was observed with regard to the route of administration, dose, tumor model and species. Regarding its use in human subjects, several data show promising results. In fact, a number of clinical trials have indicated the potential therapeutic and chemo preventive properties of resveratrol [21,131,134]. 

For example, administration of resveratrol resulted in 39% increase of the expression of cleaved caspase-3, a marker of apoptosis, in a phase I study in patients with hepatic metastases in malignant hepatic tissue, as compared with tissues from the patients treated with placebo [135], indicating a potential better response. In another study, repeated administration of Resveratrol in healthy volunteers decreased the levels of circulating IGF-I and IGFBP-3. Increased levels of these markers are associated with several types of cancer, since they have strong antiapoptotic and mitogenic properties and dysregulate cell differentiation, leading to increased neoplastic transformation and metastasis [136]. In an additional study investigating high levels of resveratrol consumption, 0.5 or 1.0 g of resveratrol administered daily before surgical resection resulted in 5% reduction of tumor cells proliferation in patients with colorectal cancer, supporting the use of resveratrol as a potential coadjuvant in specific anticancer treatments [137,138]. Finally, the effects of resveratrol on DNA methylation of cancer-related genes CCND-2, p16 and RASSF-1α were evaluated in women with high breast cancer risk. After 5–50 mg of resveratrol taken twice a day for 12 weeks, only RASSF-1 methylation was significantly reduced by resveratrol, while PGE2 (a lipid derived prostaglandin identified as a tumorigenic factor in many cancers [139]) was reduced in the nipple aspirate fluid (NAF) of the breast cancer patients that had undergone mammary ductoscopy [140]. Both these results indicated the potential beneficial effect of resveratrol in the treatment of breast cancer patients.

In fact, beside the previously described effects on p53-induction, several transcription factors implicated in cancer promotion or prevention have been shown to be affected by resveratrol, such as PPAR, PGC1α, NF-κB, Nrf2, c-Jun and CREB [141,142]. It is worth mentioning that low doses of resveratrol protected against chromosomal damage induced by four different genotoxins in both in vitro and in vivo experiments, further underscoring the efficacy of resveratrol as an agent that is able to prevent cell transformation [143]. Finally, treatment with resveratrol resulted in decreased levels of the specific oncogenic miRNAs targeting genes involved in the regulation of both tumor suppressors and effectors of the TGFβ-dependent signaling pathway [92,144,145,146,147]. 

## 2. Rapamycin

Rapamycin, also known as sirolimus, is a macrolide produced from streptomyces bacteria to inhibit fungal growth [148]. Rapamycin and its analogs inhibit the mammalian Target of Rapamycin (mTOR) complex 1 (mTORC1), an important molecule involved in cell proliferation. These drugs are currently approved by the FDA for the treatment of a number of conditions, as they have been demonstrated, both in vitro and in vivo, to slow aging, extend life span and prevent age-related diseases, including diabetic complications such as retinopathy [149].

Regarding the effect of rapamycin on the immune system, the molecule is considered an important immuno-modulator with immunosuppressive properties. mTOR (also known as FRAP1) is a major regulator of memory CD8^+^ T-cell differentiation. And contrary to what could be expected, administration of the immunosuppressive drug rapamycin results in immunostimulatory effects on the generation of memory CD8^+^ T cells, central players in the development of protective immunity. For this reason, eliciting an effective memory T-cell response is considered a major goal toward the development of functional vaccines against chronic infections and tumors. Consequently, several strategies have been employed for the improvement of vaccine strategies aimed at increasing the extent of the response relying on memory T-cells, and recently, several studies have focused on improving the functional qualities of the response (Table 2).

In this regard, it has been shown that rapamycin administered to mice intraperitoneally for about 30–40 days (75–600 μg/kg daily) following infection with acute lymphocytic choriomeningitis virus enhanced not only the amount, but also the quality/function of virus-specific CD8^+^ T cells [150]. Moreover, rapamycin (intra muscles for about 30–40 days (10–50 μg/kg daily) also increased memory T-cell responses in nonhuman primates after vaccination with modified Ankara virus, showing effectiveness during both phases of the T-cell response, namely expansion and contraction [150]: first, it increased the number of memory precursors, and second (during the effector to memory transition phase) it increased the differentiation of memory T-cell [150].

As a potential treatment against hepatitis B virus (HBV), rapamycin strongly inhibited the interaction between HBV large surface antigen (LHBs) and HBV entry receptor, sodium taurocholate cotransporting polypeptide (NTCP). This resulted in a strong reduction in hepatocyte infection with HBV with minimal cytotoxicity. The specific mechanism was identified in the direct interaction of rapamycin with NTCP, as demonstrated by surface plasmon analysis. Of note, rapamycin also prevented infection by hepatitis D virus, which also uses NTCP to enter cells [151].

Regarding Epstein-Barr virus (EBV), rapamycin inhibition of the mTOR pathway reduced total levels of BZLF1 transcripts, an immediate-early gene [152]. In addition, rapamycin alters the production of transcripts both in a cell-type, and in variant-type specific pattern, suggesting that the differential expression of these transcripts may control the replication cycle of EBV following infection [153].

Regarding HIV infection, rapamycin treatment of CD4 T cells in vitro reduces their CCR5 density levels (a coreceptor necessary for viral entry into the cells) and inhibits HIV-1 replication of CCR5-dependent virions (R5 strains). Not surprisingly, rapamycin improved the antiviral activity of T-20, an HIV-fusion inhibitor peptide used in therapy against R5 strains [154]. These data indicate that rapamycin may be used in clinical settings to increase the antiviral effect of T-20 against R5 strains, and, by extension, of other drugs targeting CCR5 [155,156,157]. Additional strategies have also been explored to exploit the combination of immune activation and the immunosuppressive properties of the mTOR inhibitor rapamycin in conjunction with T cell-activating agents in HIV-1 cure strategies [158]. Of note, rapamycin treatment did not impair cytotoxic T lymphocyte (CTL) recognition or the killing of HIV infected cells [158].

Recently, to evaluate its effect in the course of SARS-CoV-2 infection and the related cytokine storm, rapamycin (sirolimus) (6 mg on day 1 plus 2 mg daily for 13 days or until hospital discharge, whatever happens sooner) was used in clinical trials phase II in a randomized double blind placebo controlled study for the treatment of hospitalized patients with COVID-19. During the trial, several specific biomarkers were measured at day 3, 7 and 14; the study is supposed to conclude and post results soon (https://clinicaltrials.gov/ct2/show/NCT04341675).

The field of oncolytic viruses as treatment for cancer patients is currently expanding. Numerous clinical trials are ongoing, highlighting the vast clinical potential of this therapeutic tool. The current emphasis is on combining these viruses with molecules that upregulate viral replication, thus increasing oncolysis. Based on potential mechanisms by which mTOR inhibitors might enhance viral oncolysis, the antitumor effects of the combination of rapamycin and reovirus was tested in B16F10 cell, a murine model of malignant melanoma, providing evidence of synergistic antitumor cytotoxicity [159].

## 3. Metformin

Metformin is a natural compound derived from Galega officinalis. It is a widely used antihyperglycemic with a good safety profile and very few side-effects; additionally, it is inexpensive, and its usage has been associated with weight loss. Metformin is administered as an oral drug and is currently used to therapeutically treat patients affected by type-2 diabetes mellitus.

The mechanisms of action of metformin have been extensively studied [160,161,162]. Several cellular targets have been identified [163,164]; in particular, it inhibits the respiratory complex I of the electron transport chain [165,166], directly affecting reactions requiring ATP and indirectly acting on the activation of AMPK [161,164,165]. This, in turn, results in the inhibition of fatty acid synthesis and of the target of the rapamycin signaling network (mTOR) [167], causing reduced cellular energy consumption. An additional consequence of complex I inhibition is associated with increased accumulation of NADH compared to NAD+, affecting cellular biochemistry [168]. Indeed, metformin inhibits the protein NADH-ubiquinone oxidoreductase, which is localized on the mitochondrial membrane, thus activating AMPK and suppressing gluconeogenesis [169,170,171].

Metformin has also been shown to synergize with several anticancer drugs and help to reduce the chemo- and/or radio-resistance of different tumors. In addition, several data obtained both in vitro and in vivo suggest antiproliferative effects on tumor cells, and several molecular mechanisms have been described to account for these observations. In this regard, a number of clinical observations over the last few years indicate that metformin (used in doses between 0.5 and 1.5 mg/daily for at least six months) seems to reduce the risk of cancer development in diabetic patients and improve response to certain therapies and survival time in patients with certain types of cancers, such as non-small cell lung cancer [172,173,174,175], gastric cancer [176], colorectal cancer [177,178], prostate cancer [178,179], breast cancer [180,181] and pancreatic cancer [182,183]. These data, though still awaiting further validation, provide the basis for the use of metformin as an adjuvant therapy against cancer development and progression [184,185,186].

Recently, it was shown that metformin (1.7 mg/day for four months) caused important changes in the composition of the gut microbiota in diabetic patients, contributing to a better effect of the treatment [187]. In addition, in mice with endotoxemia-induced lung injury, metformin (400 mg/kg, intraperitoneal injection 30 min before LPS exposure) reduced levels of proinflammatory cytokines and improved survival [188]. These microbe-modulating properties of metformin prompted the study of several therapeutic areas of employment for metformin (Table 3).

Indeed, metformin showed efficacy as an antimicrobial agent against *Trypanosomiasis cruzi*, both in vitro and in mice (administered by gavage 400 mg/Kg/daily starting 20 days before infection and continuing for 71 days after infection) [189]. It also showed efficacy as an adjuvant in a retrospective analysis of diabetic patients with tuberculosis, and concomitantly showed that mice treated with 250 mg/kg/daily for three weeks had better outcome when infected with TB [190]. In vitro, the drug was active against *Trichinella spiralis*, *Staphylococcus aureus* and *Pseudomonas aeruginosa*, and there are indications of its potential beneficial role in sepsis, strongly suggesting that it could have a role as an agent or an adjuvant for the treatment of multiple infectious diseases [191].

When administered in combination with antiviral compounds such as interferon-α2b or Lamivudine (LMV), metformin enhanced their inhibitory effects, as measured by reduced Hepatitis B virus surface antigen (HBsAg) expression and HBV replication in vitro. An analysis of the mechanisms of action at a molecular level indicated that metformin works by partially hampering multiple HBV cis-acting elements. Based on these data, metformin could be potentially clinically helpful as an adjuvant to inhibit the HBsAg production in the treatment of HBV infection [192]. 

Infection with Hepatitis C virus (HCV) is associated with increased risk of hepatocellular carcinoma (HCC). On the other hand, treatments with metformin and statins show a protective effect. Following metformin and simvastatin treatment in vitro of human primary hepatocytes, a reduction was observed in mTOR and PTEN levels concomitant with an upregulation of levels of p62, LC3BII and Caspase 3, suggesting that this treatment could be useful in therapeutic prevention of HCV-related hepatocellular carcinoma [193].

Finally, metformin has been proposed as a potential coadjuvant treatment for COVID-19 patients [194,195,196]; this hypothesis is further supported by the fact that metformin can both reduce levels of IL-6 (important mediator of inflammation in COVID-19 patients) [197] and directly act on the pathways increasing cellular pH and subsequently interfering with the endocytic cycle, thus reducing viral replication [198,199].

## 4. Conclusions

Resveratrol, rapamycin and metformin are able to interfere with antiviral pathways and reduce viral replication and virus-related diseases, as demonstrated by experiments both in vitro and in vivo. This has prompted their use in several clinical settings. Additional studies aimed at better employing these compounds are needed, together with pharmacological strategies and dietary strategies to help strengthen their antiviral functions.

## Figures and Tables

**Table 1:** Antiviral effects of resveratrol.

Virus	Antiviral Effects
**Influenza Virus**	Block of nuclear-cytoplasmic translocation in decreased expression [31].
**Respiratory syncytial virus (RPSV)**	Reduced inflammation and levels IFN-*γ* and TLR3; inhibition of TRIF signaling, induction of M2R [35,36]; decreased production of IL-6 and TBK1 [34]; increased expression of SARM and decreased expression of MMP-12 and TRIF leading to decreased IFN-*γ* expression and AHR [33,51]; reduced levels of NGF [36]; increased levels of TNF-*α*, IFN-*γ*, and IL-2 in infected mice [32].
**Varicella Zoster virus**	Decreased synthesis of IE 62 [38].
**Epstein-Barr virus (EBV)**	Inhibition of EBV early antigen and reduced papilloma production in mouse [52]; inhibition of EBV lytic cycle resulting in reduced production of viral particles [40]; inhibition of protein synthesis, reduction in ROS production, and inhibition of transcription factors NF-κB and AP1 [53]; prevention of EBV-mediated transformation of human B-cells [39].
**Herpes simplex virus (HSV-1 and HSV-2)**	Decreased production of early viral protein ICP-4 and reduced production of viral particles; prevention of virus reactivation in latently infected neuron cells [43]; suppression of the development of cutaneous lesions in abraded skin infected with HSV-1 [44]; prevention of the development of vaginal lesions in mice infected with HSV-2 and HSV-1, with reduced mortality rate [42]; inhibition of the expression of immediate-early, early, and late HSV genes and viral DNA synthesis [41,54].
**Human immunodeficiency virus (HIV)**	Resveratrol, decitabine and 15 other derivatives of resveratrol were potent antiviral drugs [45]: inhibition of DNA synthesis [46]; block of HIV-1 infection in resting CD4 T cells; 3,3′,4,4′,5,5′-hexahydroxy-trans-stilbene (M8) showed potent anti-HIV activity [55].
**Enterovirus 71 (EV 71)**	Inhibition of viral protein 1 (VP1) synthesis and phosphorylation of proinflammatory cytokines in Rhabdomyosarcoma cell line [48].
**MERS-CoV**	Inhibition of MERS-CoV infection; downregulation of apoptosis induced by MERS-CoV in vitro [56].
**SARS-CoV-2**	Upregulation of ACE-2 [57]; decreased high levels of circulating cytokines such as IL-6 and TNF-α, upregulated following SARS-CoV-2 infection [58].

**Table 2:** Antiviral effects of rapamycin on several viruses.

Virus	Antiviral Effects
**Acute lymphocytic choriomeningitis virus**	Enhanced amount and quantity/function of virus specific CD8^+^ T cells [150].
**Hepatitis B virus (HBV)**	Inhibition of the interaction between LHBs and NCTP and consequent infection reduction [151].
**Epstein Bar Virus (EBV)**	Reduced levels of BZLF1 transcripts [152]; altered production of viral transcripts [153].
**Human immunodeficiency virus (HIV)**	Reduced CCR5 levels and improvement of antiviral compound T20 [154,155,156,157].

**Table 3:** Antibacterial and antiviral effects of metformin.

Bacterium/Virus	Effects
***T. cruzi*, *T. spiralis*, *S. aureus*, *P. aeruginosa***	Antibacterial [189,190,191]
**Hepatitis B Virus (HBV)**	Reduced HBsAg expression and viral replication [192]
**Hepatitis C Virus (HCV)**	In combination with simvastatin, it was observed reduction of mTOR and PTEN levels, and upregulation of p62, LC3BII and caspase 3 in human primary hepatocytes in vitro [193]
**SARS-CoV-2**	Reduction of IL-6 levels, increase of cellular pH levels and reduction of viral replication [194,195,196,197,198,199]
